# Assessment and simulation of eco-environmental quality changes in rapid rural urbanization: Xiong’an New Area, China

**DOI:** 10.1038/s41598-024-73487-5

**Published:** 2024-10-04

**Authors:** Zhongli Lin, Hanqiu Xu, Xiong Yao, Zhipeng Zhu

**Affiliations:** 1 College of Architecture and Urban Planning, Fujian University of Technology, Fuzhou, 350118 China; 2 College of Environmental and Safety Engineering, Fuzhou University, Fuzhou, 350116 China

**Keywords:** Eco-environmental quality assessment, Remote sensing-based ecological index, Xiong’an new area, XGBoost regression model, Multi-scenario simulation, Urban ecology, Environmental impact

## Abstract

Xiong’an New Area was established as a state-level new area in 2017 and serves as a typical representative area for studying the ecological evolution of rural areas under rapid urbanization in China. Remote sensing-based ecological index (RSEI) is a regional eco-environmental quality (EEQ) assessment index. Many studies have employed RSEI to achieve rapid, objective, and effective quantitative assessment of the spatio-temporal changes of regional EEQ. However, research that combines RSEI with machine learning algorithms to conduct multi-scenario simulation of EEQ is still relatively scarce. Therefore, this study assessed and simulated EEQ changes in Xiong’an and revealed that: (1) The large-scale construction has led to an overall decline in EEQ, with the RSEI decreasing from 0.648 in 2014 to 0.599 in 2021. (2) Through the multi-scenario simulation, the non-unidirectional evolution of RSEI during the process of urban-rural construction has been revealed, specifically characterized by a significant decline followed by a slight recovery. (3) The marginal effects of urban-rural construction features for simulated RSEI demonstrate an inverted “U-shaped” curve in the relationship between urbanization and EEQ. This indicates that urbanization and EEQ may not be absolute zero-sum. These findings can provide scientific insights for maintaining and improving the regional EEQ in urban-rural construction.

## Introduction

Today, urbanization is rapidly growing worldwide. According to the United Nations’ World Urbanization Prospects report, approximately 55% of the global population resides in urban areas^1^. It is projected that by 2050, this percentage is expected to rise to 68%, indicating not only a population shift towards urban settlements but also the development of more non-urban areas into urban built-up areas. As of 2019, China’s urbanization rate stood at 60.6%, which is significantly lower compared to the approximately 80.0% in developed countries. This highlights the substantial need for China to expand its urban spaces in the future. According to the “*The National Land Use Planning Outline*(*2016–2030*)” issued by the Central People’s Government of the People’s Republic of China in 2017 (http://www.gov.cn/zhengce/content/2017-02/04/content_5165309.htm), the urban spaces areas in China was 89,000 km^2^ in 2015. It is predicted that by 2030, this figure will increase to 116,700 km^2^.

In recent years, ecological crises have been occurring frequently worldwide, posing a serious threat to people’s lives^2–4^. Therefore, it is crucial to balance human activities and ecological environment^5^. Remarkable efforts have been actively undertaken globally for ecological conservation. Particularly in China, people have strengthened their awareness of ecological protection. Moreover, ecological security has also been elevated to the level of national strategy^6,7^. Compared to natural space, urban space is characterized by high energy consumption, high levels of pollution, and a low presence of natural resources. These characteristics contribute to the complexity, fragility, and instability of urban ecosystems^8^. Some fast-growing cities have experienced a series of ecological and environmental issues, including intensified urban heat island effects, degraded air quality, biodiversity loss, and habitat fragmentation^9,10^. Therefore, it is crucial to have timely monitoring of the ecological status in urban areas and conduct detailed assessments of past and present eco-environmental quality (EEQ). Additionally, conducting the multi-scenario simulation for regions that will undergo large-scale construction in the future is equally important.

With the rapid development of satellite-based Earth observation system, the use of remote sensing technology with its multi-scale, multi-view and near real-time capabilities offers significant advantages in monitoring and evaluating land surface ecological status from global to local scales^11–13^. Xu^14^ proposed a completely remote sensing-based ecological index (RSEI). RSEI combined four indicators that are closely related to regional ecological conditions: greenness, wetness, heat, and dryness. Principal component analysis (PCA) is used to scientifically and objectively determine the weight of each indicator, avoiding subjective weight assignment^14^. Moreover, the availability of relevant basic data for producing RSEI is high. Therefore, RSEI has been widely applied in the quantitative assessment of ecological status in different geographical environment, including cities^15,16^, rural areas^12,17^, islands^18^, forests^19^, and wetlands^20^. The results have demonstrated that RSEI can provide a fairly accurate evaluation of ecological status changes during the process of regional development.

In the research of urban and regional development, multi-scenario simulation of future development has always been one of the research hotspots. Many studies present land use and land cover evolutions as the primary outcomes, conducting multi-scenario simulation of urban development. Among these studies, the most commonly used simulation model is cellular automata (CA). CA is a grid dynamic modeling framework that incorporates discrete time, space, and state components. It can effectively simulate the spatial-temporal evolution of a complex system^21,22^. For example, Kiavarz et al.^23^ used land cover maps from 1985 to 2019 to simulate the land cover outcomes for 2026, 2032, and 2038 through the CA-Markov model, to further predict the future spatial and temporal changes of surface urban heat islands. Beyond CA, numerous studies also leveraged regression models, utilizing various variable parameters to represent distinct urban or regional development scenarios and calculate predictions for each of these scenarios. For instance, Massaro et al.^24^ employed the spatial lag model to assess the exposure of urban population to extreme land surface temperature (LST) for 200 cities worldwide through the normalized difference vegetation index in a multi-gradient and multi-scenario simulations. Their study demonstrates that multi-scenario modeling can effectively investigate the impact of extreme LST on urban residents, and achieve precisely targeting vegetation greening at an optimal cost. Xu et al.^12^ used population and impervious surface area as indicators for setting up multi-scenario in study area, and predicted the changes in RSEI under different combinations of population and impervious surface through a linear regression model. The findings indicated that alterations in the proportions of impervious surface within the new area could result in a substantial change in RSEI. Meanwhile, the study estimated an optimal total area of impervious surface in the study region to achieve a balance state under the current planning framework.

Xiong’an New Area is a state-level new area established in China, since April 2017 (http://xiongan.gov.cn/index.htm). The rapid and continuous infrastructure construction in Xiong’an New Area has resulted in significant changes in the urban and rural landscape, posing a major challenge to its ecological environment. Furthermore, the establishment of this new area carries tremendous strategic significance for region and even China’s future development. Consequently, numerous studies have been conducted on the EEQ of Xiong’an New Area. However, to our knowledge, most current studies have primarily focused on presenting the historical and current spatial-temporal variations in EEQ resulting from the region’s rapid development^25^, while research on multi-scenario simulation for future of the new area remain scarce. Additionally, there is insufficient exploration of the complex non-linear relationships between urban-rural construction features and EEQ.

Therefore, this study focused on Xiong’an New Area to quantitatively assess its EEQ using RSEI and simulate the evolution of RSEI based on XGBoost machine learning algorithm. The objectives of this study were to (1) evaluate the changes in RSEI before and after the establishment of new area, (2) build a regression model between RSEI and urban-rural construction features using XGBoost algorithm, set up the simulation scenarios with a progressive 5% change in these construction features, thereby revealing the characteristics of RSEI evolution, and (3) explore the relationship between urban construction features and RSEI, analyze the underlying causes of their formation, and investigate the potential for the co-development of regional construction and EEQ. We hope that the findings of this study will provide scientific insights for maintaining and improving the EEQ in regional urban-rural construction. It holds important practical significance for vast rural areas in exploring the eco-friendly development path.

## Study area and datasets

### Study area

**Fig. 1:**
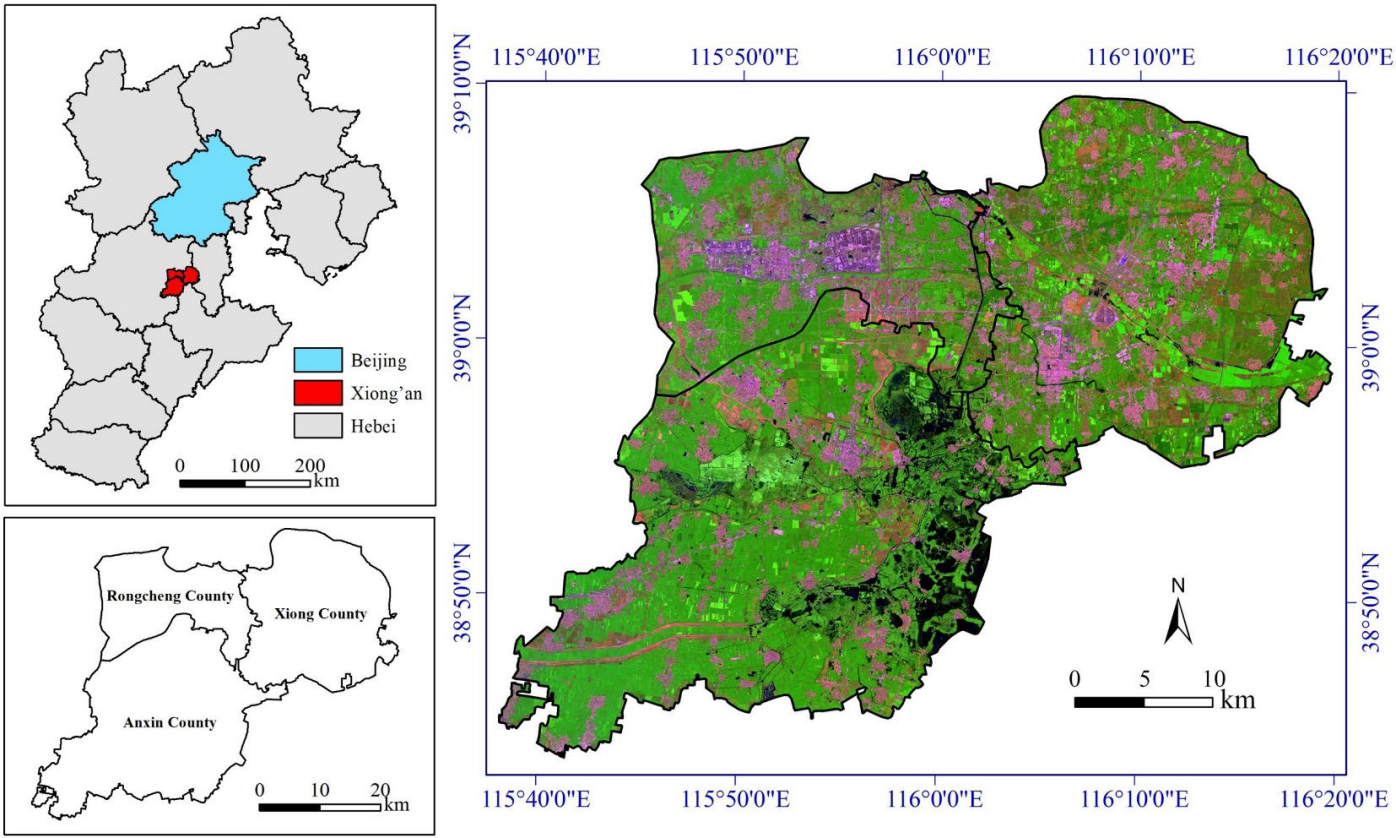
Location of Xiong’an New Area and its 2021 Landsat-8 image (09/07/2021). This map was produced using ArcGIS Version 10.7 (https://www.esri.com/en-us/arcgis/geospatial-platform/overview).

Xiong’an New Area (38°43′∼39°10′N, 115°38′∼116°20′E) includes three counties (Xiong, Rongcheng, and Anxin) and some surrounding areas (Fig. 1). It is located in the hinterland of Hebei Province, approximately 105 km from the capital city Beijing and only 55 km from Beijing Daxing International Airport. Xiong’an New Area has obvious geographical advantages and convenient transportation. The main purpose of establishing Xiong’an New Area is to divert the “non-core” functions of Beijing, the capital city of China. It aims to plan and construct a modern and innovative urban area guided by new development concepts, and to create a model for the integrated development of urban and rural areas in the new era of China. Xiong’an holds a great significance for the subsequent construction of new urban-rural areas in China.

Xiong’an New Area has a deposition plain landform, with the terrain gradually decreasing from northwest to southeast and the ground elevation ranging from 5 to 26 m. The region features a warm temperature monsoon climate with a semi-humid and semi-arid characteristic. It experiences limited rainfall in spring, abundant rainfall in summer, cold and dry weather in autumn, and little snow in winter. The average annual temperature is 12.4 °C, and the average annual precipitation is 495.1 mm. The area is crisscrossed by rivers and canals, with a well-developed water system and numerous lakes. Xiong’an New Area boasts an excellent ecological environment, abundant agricultural resources, a strong environmental carrying capacity, and ample development space. According to the data from China’s Seventh National Population Census in 2020, Xiong’an New Area had a permanent population of 1.21 million, with 478,553 residing in Xiong County, 273,164 in Rongcheng County, and 453,723 in Anxin County^26^. This research took the area covered by these three counties as study area of Xiong’an New Area, with an approximate area of 1,556.61 km^2^ (Fig. 1).

## Datasets

Lansdat-8 images, along with population density, building rooftop area, and building height datasets, were adopted as data sources (Table 1). In Xiong’an New Area, there is a large area of arable land, and agricultural crops will undergo a harvest from late September to October. After the harvest, the land surface cover mainly consists of bare soil. The extensive expanse of exposed farmland bare soil can complicate the assessment of ecological effects of regional construction. Therefore, in order to ensure that the changes in land surface coverage characteristics are primarily caused by urban construction, we limited the time frame for the study years to September when crops have not been extensively harvested. We selected Landsat-8 images for five dates in September, namely September 4, 2014, September 28, 2017, September 2, 2019, September 4, 2020, and September 7, 2021. Landsat-8 is equipped with two sensors, the spectral Operational Land Imager (OLI) and the Thermal Infrared Sensor (TIRS), with spatial resolutions of 30 m and 100 m, respectively. In this study, Landsat-8 imagery was used to retrieve land covers features and LST, and assess ecological status of the Xiong’an New Area. The population density data was derived from WorldPop website (https://www.worldpop.org/), which is a global high resolution population denominators project. WorldPop provides the open access spatial demographic datasets. We utilized the population spatial data for China in 2020, which estimates the total number of people per grid cell at a resolution of 3 arc seconds (approximately 100 m at the equator)^27^. The building density data was derived from China Building Rooftop Area (CBRA) dataset. CBRA is the first multi-annual (2016–2021) and full-coverage building rooftop area dataset in China, with 2.5 m spatial resolution. It achieves good performance with an overall accuracy of 82.85%^28^. The building height data was derived from the first Chinese building height map at 10 m resolution (CNBH-10 m). CNBH-10 m was based on all-weather earth observations data and used the random forest model to estimate the 2020 building height in China. The root mean square error (RMSE) and mean absolute error (MAE) of CNBH-10 m were 6.1 m and 5.2 m, respectively^29^.

**Table 1:** Datasets used in this study.

Data	Resolution	Date	Usage	Data source
Landsat-8 images	30 m (spectral bands of OLI sensor); 100 m (thermal bands of TIRS)	09/04/2014 09/28/2017 09/02/2019 09/04/2020 09/07/2021	Retrieval of land covers features and land surface temperature, and assessment of ecological effects	https://earthexplorer.usgs.gov/
WorldPop dataset	100 m	2020	Spatial distribution of population density	https://www.worldpop.org/datacatalog/
China building rooftop area dataset	2.5 m	2021	Spatial distribution of building density	https://zenodo.org/record/7500612
Chinese building height dataset	10 m	2020	Spatial distribution of building height	https://zenodo.org/record/7064268#.ZEQM-vxByUm

## Methods

### RSEI ecological index

RSEI is a regional EEQ assessment index that is completely based on remote sensing technology. Compared to traditional ecological indices, the most notable improvement in RSEI is its selection of four important indicators, namely greenness, wetness, heat, and dryness, which directly reflect the ecological quality^14^. These indicators are used for dimensionality reduction calculation, which not only avoid one-sided evaluation of a single indicator, but also integrate the information from multiple indicators into a concise visual index or result. The functional form of RSEI can be expressed as:

1 $$RSEI = f\left( {Greenness,Wetness,Heat,Dryness} \right)$$

NDVI is capable of reliably assessing vegetation quality and productivity, and it has the advantages of being concise and easy to calculate^30^. NDVI is currently the most widely used remote sensing vegetation index. In this study, greenness indicator in RSEI is represented by NDVI. NDVI is expressed as:

2 $$NDVI = \left( {NIR - Red} \right)/\left( {NIR + Red} \right)$$

where *NIR* and *Red* are the near-infrared (NIR) band and red band of Landsat-8 reflectance images, respectively.

Tasseled cap transformation (TCT) is a valuable tool for compressing spectral data into a few components that correspond to the physical characteristics of a scene, while minimizing information loss^31^. Among them, the wetness component is associated with soil and plant moistures, water and other moist features^32^. We used wetness component to characterize the wetness indicator in RSEI. The wetness component of TCT is expressed as^31^:

3 $$wetness = 0.1511Blue + 0.1973Green + 0.3283Red + 0.3407NIR - 0.7117SWIR1 - 0.4559SWIR2$$

where *Blue*, *Green*, *Red*, *NIR*, *SWIR*1 and *SWIR*2 corresponding to the blue, green, red, NIR, short-infrared 1 (SWIR1), and short-infrared 2 (SWIR2) bands of Landsat-8 reflectance images, respectively.

Dryness indicator is mainly used to quantitatively depict the “dryness” characteristics on the land surface caused by the replacement of natural cover with artificial surface due to human activities in a given region. In studies focusing on urban built-up area, commonly used indices such as the building/built-up index or the impervious surface index were employed to represent this indicator. However, unlike urban built-up areas, Xiong’an New Area is located in rural areas. Apart from the rural settlements, there are currently large areas of bare soil in this region, which are primarily caused by land leveling activities during the early stages of engineering construction and crop harvesting. In this study, therefore, the normalized difference impervious surface index (NDISI)^33^ and the normalized difference soil index (NDSI)^34^ were combined to construct the normalized difference impervious surface and soil index (NDISSI) to represent the Dryness indicator in RSEI. The NDISSI is expressed as:

4 $$NDISSI = \left( {NDISI_{{{\text{nor}}}} + NDSI_{{{\text{nor}}}} } \right)/2$$

where *NDISI*_nor_ and *NDSI*_nor_ are normalized NDISI and NDSI, respectively. NDISI and NDSI are expressed as:

5 $$NDISI = \frac{{TIR - \left[ {\left( {WI + NIR + SWIR1} \right)/3} \right]}}{{TIR + \left[ {\left( {WI + NIR + SWIR1} \right)/3} \right]}}$$

6 $$NDSI = \left( {SWIR1 - NIR} \right)/\left( {SWIR1 + NIR} \right)$$

where *TIR* is the thermal band; *WI* represents water index-derived band and can be represented by the modification of normalized difference water index (MNDWI)^35^.

The normalization algorithm can be expressed as:

7 $$I_{{{\text{nor}}}} = \frac{{I_{i} - I_{{\min }} }}{{I_{{\max }} - I_{{\min }} }}$$

where *I*_nor_ is the normalized index or indicator; *I*_*i*_ represents the value of the index or indicator in pixel *i*; *I*_*max*_ and *I*_*min*_ represent the maximum and minimum values of the index or indicator, respectively.

Heat indicator in RSEI is represented by LST. In this study, Landsat-8 TIRS band 10 was utilized. The single channel (SC) algorithm proposed by Jiménez-Muñoz and Sobrino^36^, Jiménez-Muñoz et al.^37^, and Cristóbal et al.^38^ was adopted to retrieve LST. The SC algorithm is expressed as follows:

8 $$LST = \gamma \times \left[ {\varepsilon ^{{ - 1}} \times \left( {\psi _{1} \times L + \psi _{2} } \right) + \psi _{3} } \right] + \delta$$

9 $$\gamma = T^{2} /\left( {b_{\gamma } \times L} \right)$$

10 $$\delta \approx T - T^{2} /b_{\gamma }$$

where *L* is the at-sensor spectral radiance of TIRS band 10; *ε* is the land surface emissivity; *γ* and *δ* are two parameters dependent on Planck’s function; *b*_*γ*_ = 1324 for TIRS band 10; *ψ*_1_, *ψ*_2_, and *ψ*_3_ are the atmospheric functions calculated as:

11 $$\psi _{1} = 1/\tau ,\psi _{2} = - L^{ \downarrow } - L^{ \uparrow } /\tau ,\psi _{3} = L^{ \uparrow }$$

where *τ* is the atmospheric transmissivity, and *L*^↑^ and *L*^↓^ are the upwelling and downwelling atmospheric radiance, respectively. *T* is the at-sensor brightness temperature calculated as follows:

12 $$T = K_{2} /\ln \left( {K_{1} /L + 1} \right)$$

where *K*_1_ and *K*_2_ are the band-specific thermal conversion constants, *K*_1_ = 774.89 (W/m^2^/sr/µm) and *K*_2_ = 1321.08 K for TIRS band 10.

The PCA algorithm, a multivariate statistical method, was utilized in the construction of RSEI. Through rotating the coordinate axes of feature spectral space to maximize the removal of correlation among different indicators, the key information of multivariate is concentrated into the first few principal components, such as the first principal component (PC1). The advantage of PCA lies in its utilization of covariance matrix for automatic and objective calculation of the weight distribution for each indicator, and in the objective determination of the contribution of each principal component through the corresponding eigenvalue. Any weighting structure is susceptible to criticism, as assigning weights is a subjective value-dependent process^39^. While, PCA effectively avoids result biases caused by arbitrary subjective weighting of indicators^14^. The RSEI function in Eq. (1) can be further divided into the following two steps:

13 $$RSEI_{0} = 1 - {\text{PC}}1\left[ {f\left( {NDVI,Wetness,LST,NDISSI} \right)} \right]$$

14 $$RSEI = RSEI_{{0\:{\text{nor}}}}$$

It is important to note that due to the different units of measurement among indicators, it is necessary to normalize each indicator using Eq. (7) before performing PCA. The normalization process ensures that all the indicators are on a unified scale within the range of 0 to 1. Additionally, if there are large water bodies in the study area, they may have an impact on the analysis of indicators loading in PCA^14^. In such cases, the MNDWI can be used to mask out the water information. The resulting RSEI is referred to as the normalized *RSEI*_0_ (*RSEI*_0 nor_), which has value range from 0 to 1. A higher value of RSEI, that is closer to 1, indicates a better EEQ, while a value of 0 denotes an extremely poor one.

### XGBoost algorithm

The XGBoost algorithm, initially proposed by Chen et al.^40^, is an optimized distributed gradient boosting library that has found extensive applications in various fields, including the built environment^41,42^, natural geography^43^, and agricultural and biological^44^. It has demonstrated high accuracy in these domains. As a ensemble decision trees, XGBoost initializes with a base prediction and then iteratively constructs trees to fit the residuals (i.e., the differences between the observed and predicted values) from the previous iterations. This process repeats multiple times, allowing XGBoost to achieve highly accurate predictions^45^. One notable advantage of XGBoost is its ability to refine its predictions through multiple rounds of iterations over the residuals^42^.

XGBoost is a highly scalable machine learning system developed from the concept of Gradient Boost Decision Tree (GBDT)^43^, with multiple adjusted hyperparameters. Configuring these hyperparameters appropriately is a crucial step in achieving model accuracy. In this study, the seven hyperparameters, namely eta, gamma, max_depth, min_child_weight, subsample, colsample_bytree, and nrounds, were iteratively calculated within a preset hyperparameters tuning space (Table 2). A 5-fold cross validation approach^46^ was employed to determine the optimal hyperparameters combination that minimizes the model’s error. Furthermore, the Shapley Additive exPlanation (SHAP) analysis is based on game theory and relies on SHAP values to determine the importance of individual independent variables^47^. It takes into account their marginal contribution to the XGBoost regression model outcome.

**Table 2:** The hyperparameters of the XGBoost algorithm used in this study and their corresponding tuning space.

Hyperparameter	Description	Default value	Hyperparameter tuning space preset in this study
eta	Learning rate used to prevent overfitting by making the boosting process more conservative	0.3	[0, 1]
gamma	Minimum loss reduction required to make a further partition on a leaf node of the tree	0	[0, 20]
max_depth	Maximum depth of a tree	6	[1, 20]
min_child_weight	Minimum sum of instance weight (hessian) needed in a child	1	[1, 10]
subsample	Subsample ratio of the training instances	1	[0.5, 1]
colsample_bytree	subsample ratio of columns when constructing each tree	1	[0.5, 1]
nrounds	Max number of boosting iterations	100	[50, 300]

### Multi-scenario simulation

**Fig. 2:**
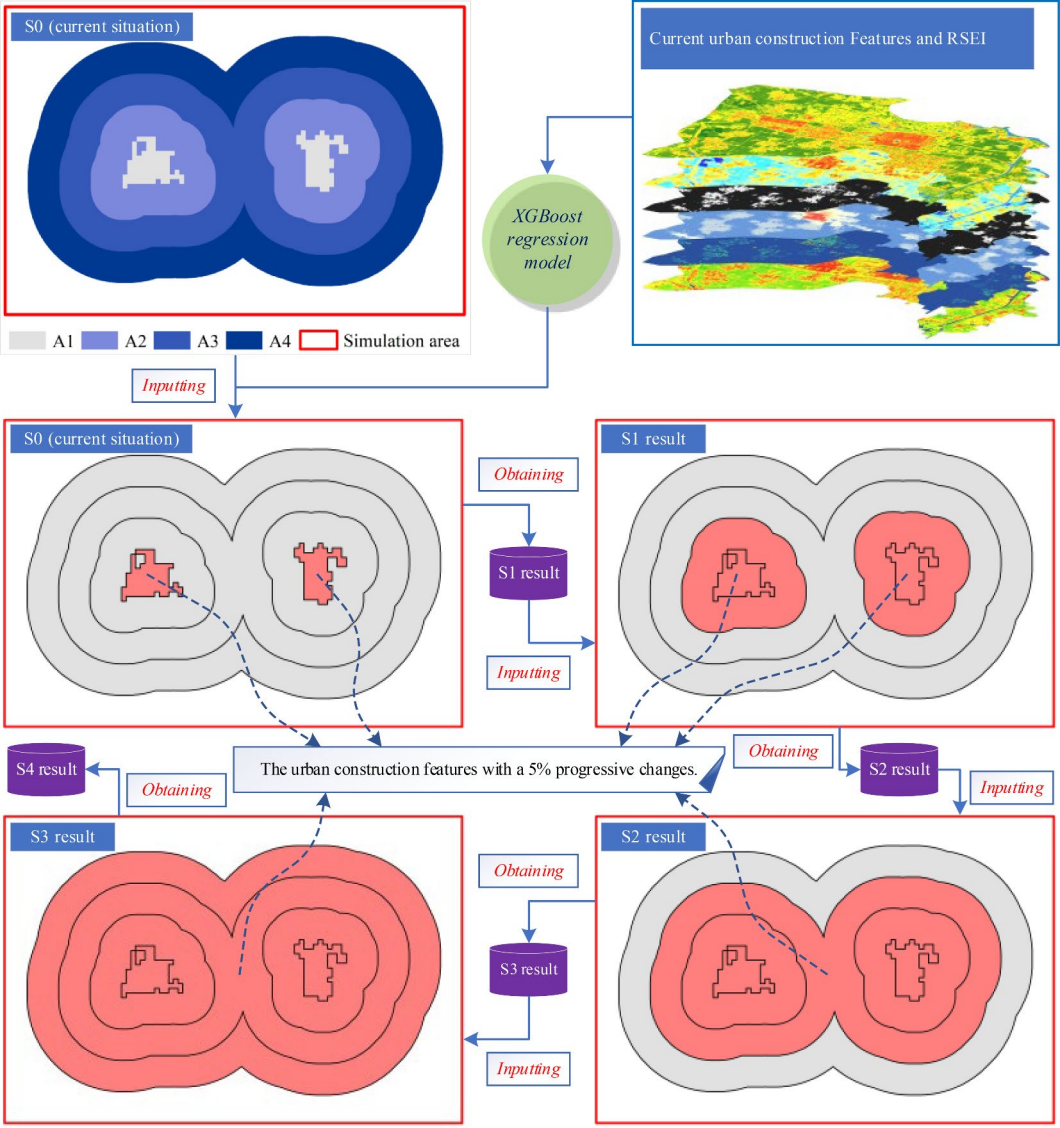
Illustration of multi-scenario simulation workflow.

This study was based on the 2021 conditions of study area as the basis for multi-scenario simulation. The illustration of multi-scenario simulation workflow is shown in Fig. 2. First, the built-up areas and bare soil areas under development within the study area were extracted using the NDISSI threshold, forming the initial region A1. Then, concentric buffer zones of 200 m, 400 m, and 600 m were created around A1, forming buffer regions A2, A3, A4, respectively. Next, relevant urban-rural construction features of the current study area in 2021 (S0) was obtained, including NDVI_nor_, NDISI_nor_, percentage of the building rooftop (PB), population density (PopD), and building height (BH). These construction features were then progressively changed by 5% starting from region A1 with a step length of 200 m to simulate the possibility of urban construction in multiple scenarios. Four scenarios were simulated as follows: Scenario One (S1) involved a 5% progressive change in the urban-rural construction features of region A1 based on current situation (S0), specifically a decrease of 5% in NDVI_nor_ and an increase of 5% in NDISI_nor_, PB, PopD, and BH, while other non-A1 regions remain unchanged. The modified urban-rural construction features were used in the XGBoost regression model to simulate the RSEI, resulting in S1. Similarly, Scenario Two (S2) was built upon S1 with a progressive change of 5% in the construction features for regions A1 and A2. Likewise, Scenario Three (S3) was based on S2 and involved a change of 5% in the construction features for regions A1, A2, and A3. Finally, Scenario Four (S4) was built upon S3 with a change of 5% in the construction features for regions A1, A2, A3, and A4.

It is worth noting that during the simulation, if NDVI_nor_ falls below 0, it is adjusted to 0. If NDISI_nor_ and PB exceed values of 1 or 100%, they are constrained to 1 or 100% accordingly. Additionally, in the newly added buffer region, if PopD and BH are both 0, the minimum non-zone values within that region are utilized as substitute.

## Results

### Construction of RSEI

Table 3 presents the PCA results of four indicators (greenness, wetness, heat, and dryness) in 2014, 2017, 2019, 2020, and 2021. It can be observed that PC1 has the highest loading in PCA of the four indicators for the five study years, accounting for 92.59%, 84.62%, 93.16%, 92.59%, and 90.67%, respectively. Additionally, the coefficients of four indicators in PC1 exhibit stable patterns, with negative values for greenness and wetness, and positive values for heat and dryness. However, PC2–4 not only have significantly lower loadings, but also the signs of their coefficients vary inconsistently across different years, indicating a lack of stability. This suggests that PC1 effectively represents the comprehensive information of greenness, wetness, heat, and dryness.

**Table 3:** Principal component analysis (PCA) of four indicators for Xiong’an New Area.

	PC1	PC2	PC3	PC4
2014				
Greenness	− 0.73	0.68	− 0.10	0.05
Wetness	− 0.20	− 0.22	− 0.44	− 0.85
Heat	0.40	0.29	− 0.83	0.27
Dryness	0.53	0.64	0.32	− 0.46
Covariance eigenvalue	0.075	0.003	0.002	0.001
Percent covariance eigenvalue (%)	92.59	3.70	2.47	1.23
2017				
Greenness	− 0.68	0.71	0.08	− 0.19
Wetness	− 0.40	− 0.33	− 0.85	− 0.14
Heat	0.28	0.50	− 0.44	0.70
Dryness	0.55	0.38	− 0.30	− 0.68
Covariance eigenvalue	0.044	0.005	0.002	0.001
Percent covariance eigenvalue (%)	84.62	9.62	3.85	1.92
2019				
Greenness	− 0.60	0.79	0.13	0.03
Wetness	− 0.25	− 0.05	− 0.74	− 0.62
Heat	0.55	0.49	− 0.55	0.39
Dryness	0.53	0.37	0.37	− 0.68
Covariance eigenvalue	0.109	0.005	0.002	0.001
Percent covariance eigenvalue (%)	93.16	4.27	1.71	0.85
2020				
Greenness	− 0.65	− 0.64	0.37	− 0.18
Wetness	− 0.42	− 0.02	− 0.88	− 0.23
Heat	0.24	− 0.60	− 0.28	0.71
Dryness	0.59	− 0.48	− 0.10	− 0.64
Covariance eigenvalue	0.050	0.002	0.002	0.000
Percent covariance eigenvalue (%)	92.59	3.70	3.70	0.00
2021				
Greenness	− 0.55	0.45	0.68	− 0.19
Wetness	− 0.31	− 0.62	− 0.06	− 0.71
Heat	0.43	− 0.50	0.73	0.18
Dryness	0.64	0.41	0.07	− 0.65
Covariance eigenvalue	0.068	0.003	0.003	0.001
Percent covariance eigenvalue (%)	90.67	4.00	4.00	1.33

According to Eqs. (13) and (14), it can be observed that in the calculation of RSEI_0_, the sign of RSEI_0_ is exactly opposite to that of PC1. Hence, the sign of RSEI is also opposite to that of PC1. This implies that greenness and wetness have a positive effect on regional EEQ, while heat and dryness lead to a decline in EEQ. Moreover, by examining the absolute value of the coefficients of the four indicators in PC1, it is noted that greenness has the highest loading of 0.73 in 2014, indicating its most significant impact on regional ecology. However, by 2021, the loading of greenness decreases to 0.55, while dryness, the indicator representing regional construction intensity, takes its place with a loading of 0.64. This shift in the influence indicators suggests that after Xiong’an became a state-level new area, its natural land surface was inevitably replaced by large-scale construction land, leading to changes in key indicators. Specifically, the loading of greenness, which had a positive effect on EEQ, continuously decreased, while the loading of dryness, which had a negative effect on EEQ, kept increasing.

### Spatiotemporal distributions of RSEI

**Fig. 3:**
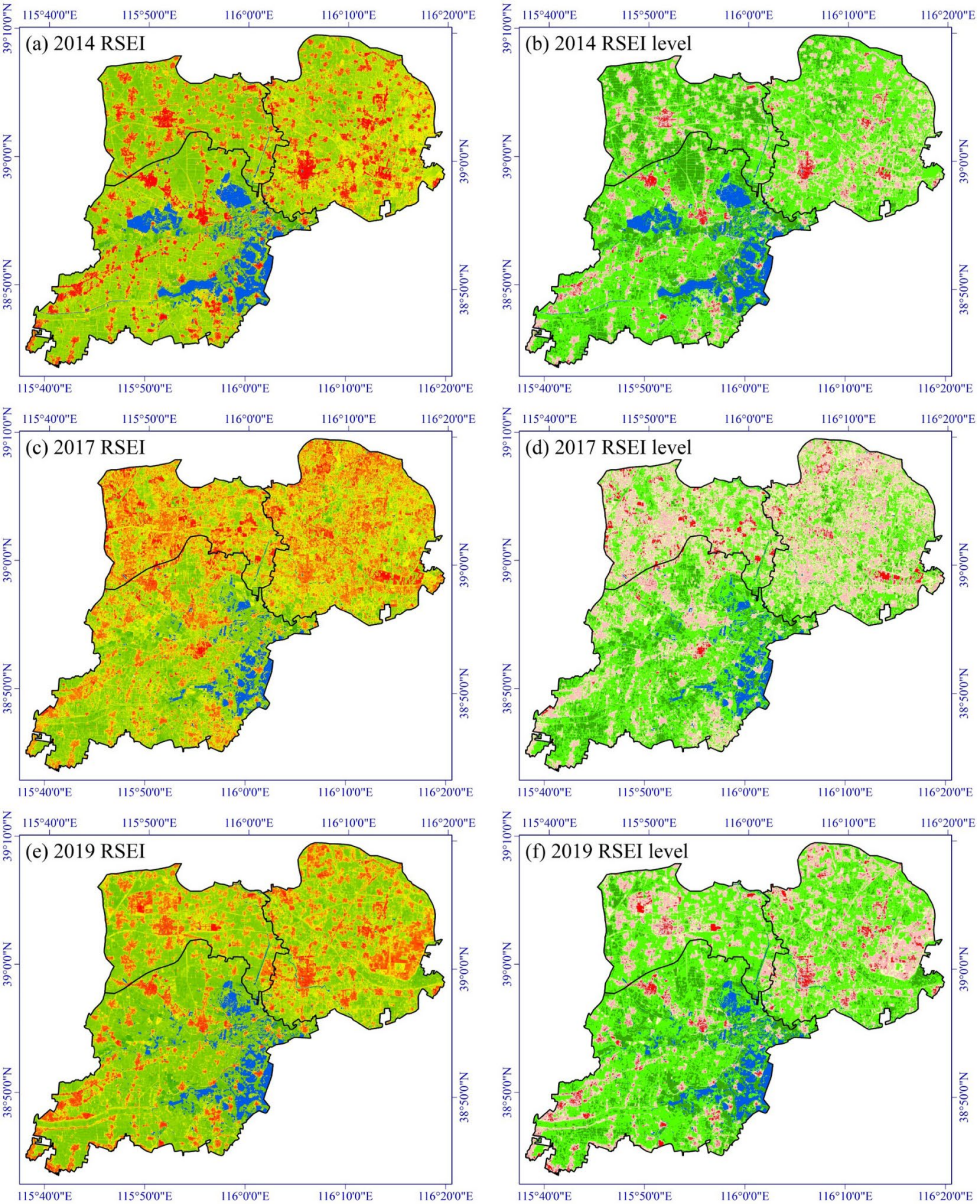

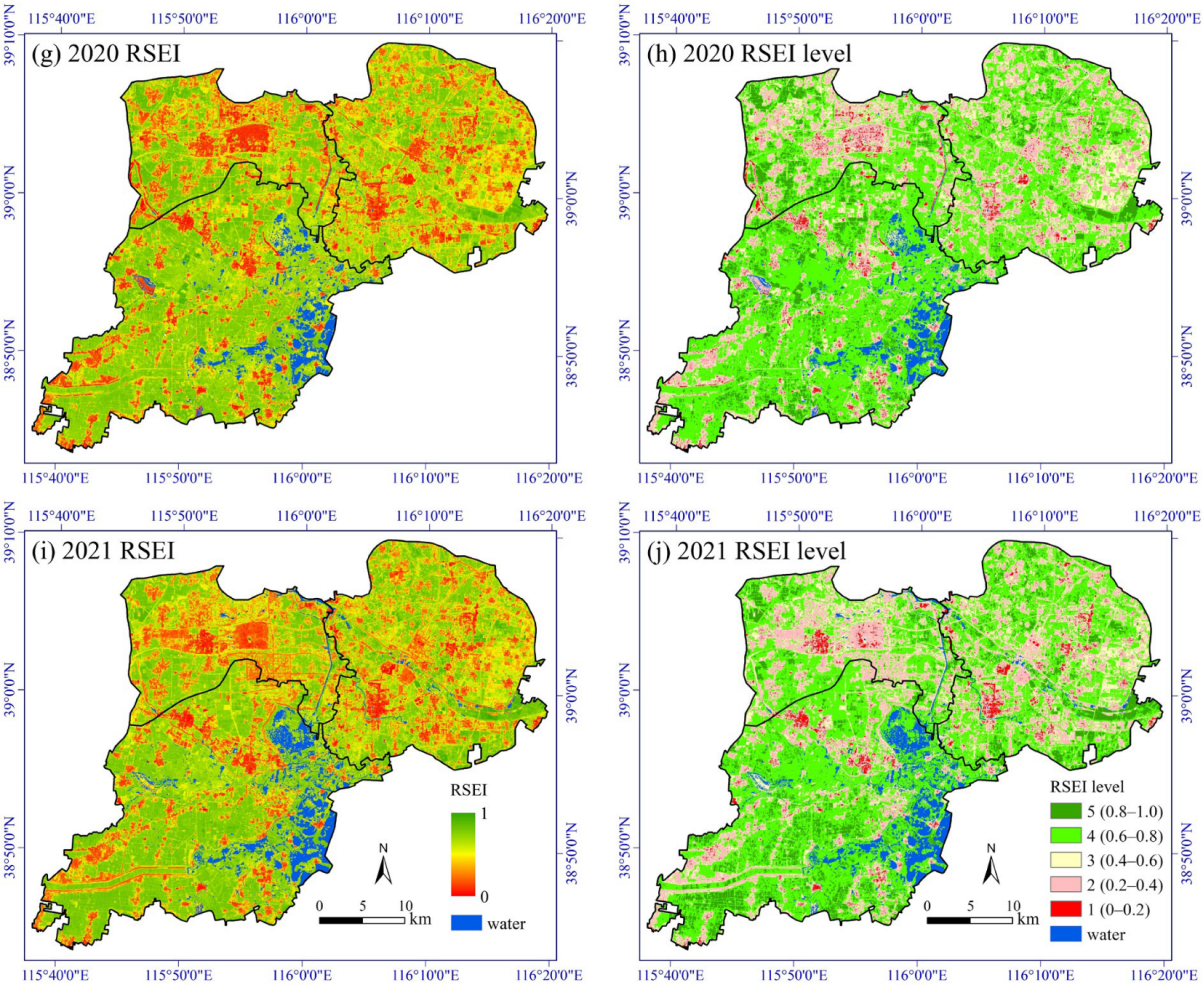
Spatiotemporal distribution of RSEI and RSEI level in Xiong’an New Area. This map was produced using ArcGIS Version 10.7 (https://www.esri.com/en-us/arcgis/geospatial-platform/overview).

Figure 3a, c, e, g, i show the spatial distribution of RSEI in Xiong’an New Area in 2014, 2017, 2019, 2020, and 2021, respectively. We further classified RSEI into five levels with an interval of 0.2 (Fig. 3b, d, f, h, j). Level 5 represents excellent EEQ, while Level 1 denotes extremely poor one, i.e., Level 5: 0.8–1.0; Level 4: 0.6–0.8; Level 3: 0.4–0.6; Level 2: 0.2–0.4; Level 1: 0–0.2. Therefore, the final RSEI score represents the degree of naturalness of Xiong’an New Area.

**Fig. 4:**
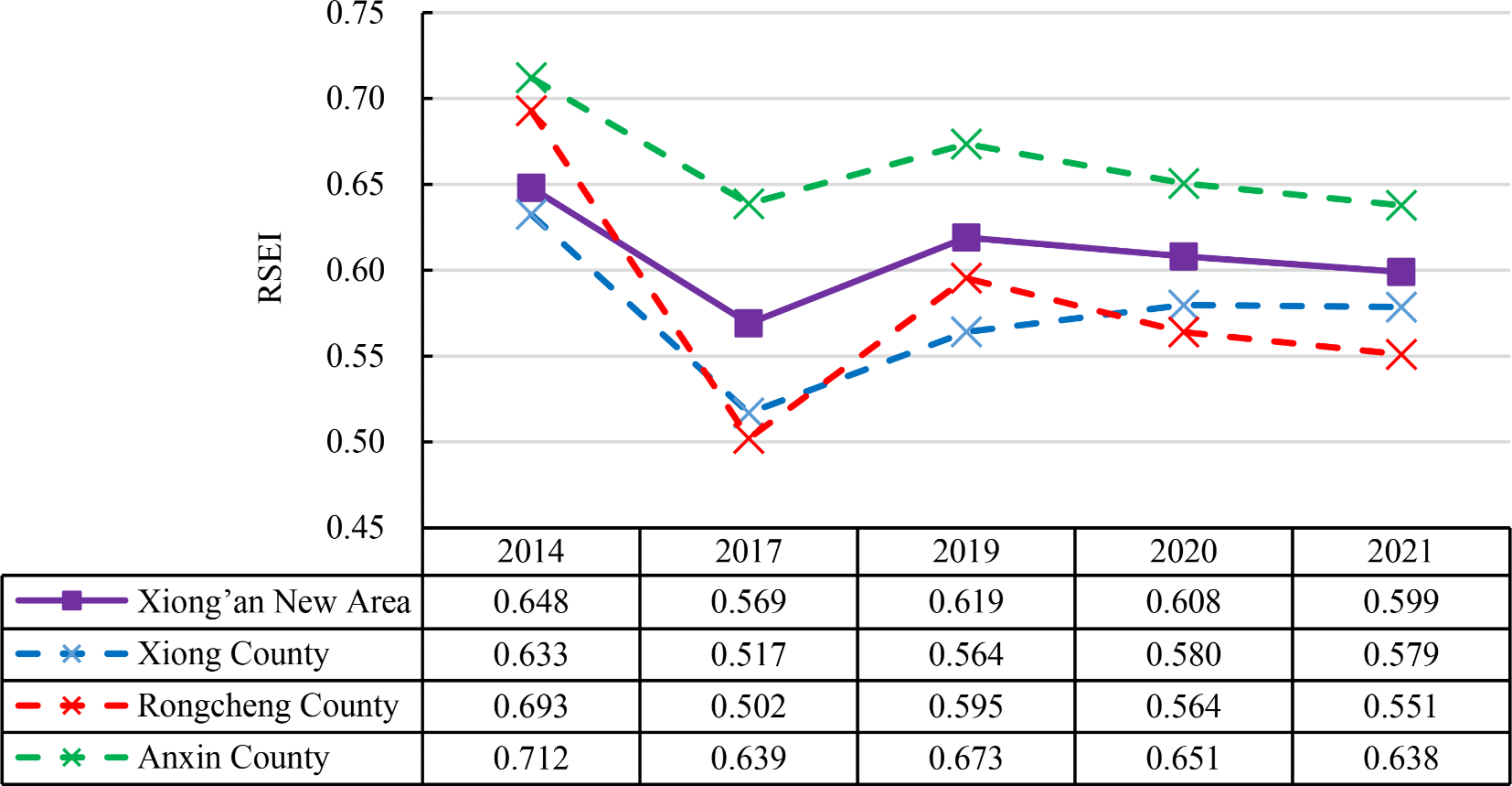
Changes in RSEI results for Xiong’an New Area and its three counties.

In terms of overall RSEI results, Xiong’an New Area has a solid ecological foundation, with dense arable land and well-developed water system. The higher ecological levels, Level 4–5, mainly consist of the cultivated land covered by mature crops and natural land surface such as trees and grasslands (Fig. 3). From a temporal perspective, EEQ exhibits an overall downward trend from 2014 to 2021, with RSEI decreasing from 0.648 in 2014 to 0.619 in 2019, and further to 0.608 in 2020 and 0.599 in 2021 (Fig. 4). However, it can also be observed that RSEI experienced a fluctuation outside the overall trend in 2017, when it dropped to 0.569, the lowest value among the five study years. This is mainly because a widespread area of bare soil was found in 2017, which could be attributed to the harvesting of some crops at the end of September, based on the image acquisition data of September 28th. According to the results of RSEI and RSEI level (Fig. 3), the low-value areas of RSEI (Level 1–2) are primarily found in rural settlements. However, in 2017, due to the emergence of a large amount of bare soil, Level 1–2 patches appear to be more dispersed. Since the decrease in RSEI caused by crop harvesting is temporary and cyclical, RSEI will rise again with the replanting and growth of crops. Therefore, the downward fluctuation of RSEI in 2017 is not due to the decline of EEQ associated with urbanization.

Table 4 shows the statistics of RSEI level in Xiong’an New Area. It can be seen that from 2014 to 2021, the RSEI level that experienced the greatest change is Level 5, which represents the best ecological status. Its percentages sharply decreased from 17.82% in 2014 to 10.25% in 2021, a decrease of 7.57%. Meanwhile, Level 2, representing poorer ecological status, increased from 13.82% in 2014 to 20.21% in 2021, an increase of 6.39%.

**Table 4:** RSEI level statistics of Xiong’an New Area.

RSEI level	2014		2017		2019	
	Area (km^2^)	Percentage (%)	Area (km^2^)	Percentage (%)	Area (km^2^)	Percentage (%)
5 (0.8–1.0)	259.85	17.82	135.59	8.97	187.95	12.66
4 (0.6–0.8)	774.13	53.10	612.28	40.52	787.70	53.07
3 (0.4–0.6)	194.59	13.35	402.88	26.66	202.90	13.67
2 (0.2–0.4)	201.56	13.82	327.76	21.69	263.50	17.75
1 (0–0.2)	27.85	1.91	32.47	2.15	42.26	2.85
Σ	1457.98	100.00	1510.98	100.00	1484.31	100.00

RSEI level	2020		2021		Difference between 2014 and 2021	
	Area (km^2^)	Percentage (%)	Area (km^2^)	Percentage (%)	Percentage (%)	
5 (0.8–1.0)	140.14	9.33	151.08	10.25	− 7.57	
4 (0.6–0.8)	802.38	53.43	742.41	50.34	− 2.76	
3 (0.4–0.6)	265.26	17.66	250.21	16.97	3.62	
2 (0.2–0.4)	262.08	17.45	298.03	20.21	6.39	
1 (0–0.2)	31.87	2.12	32.96	2.24	0.33	
Σ	1501.73	100.00	1474.70	100.00	–	

**Fig. 5:**
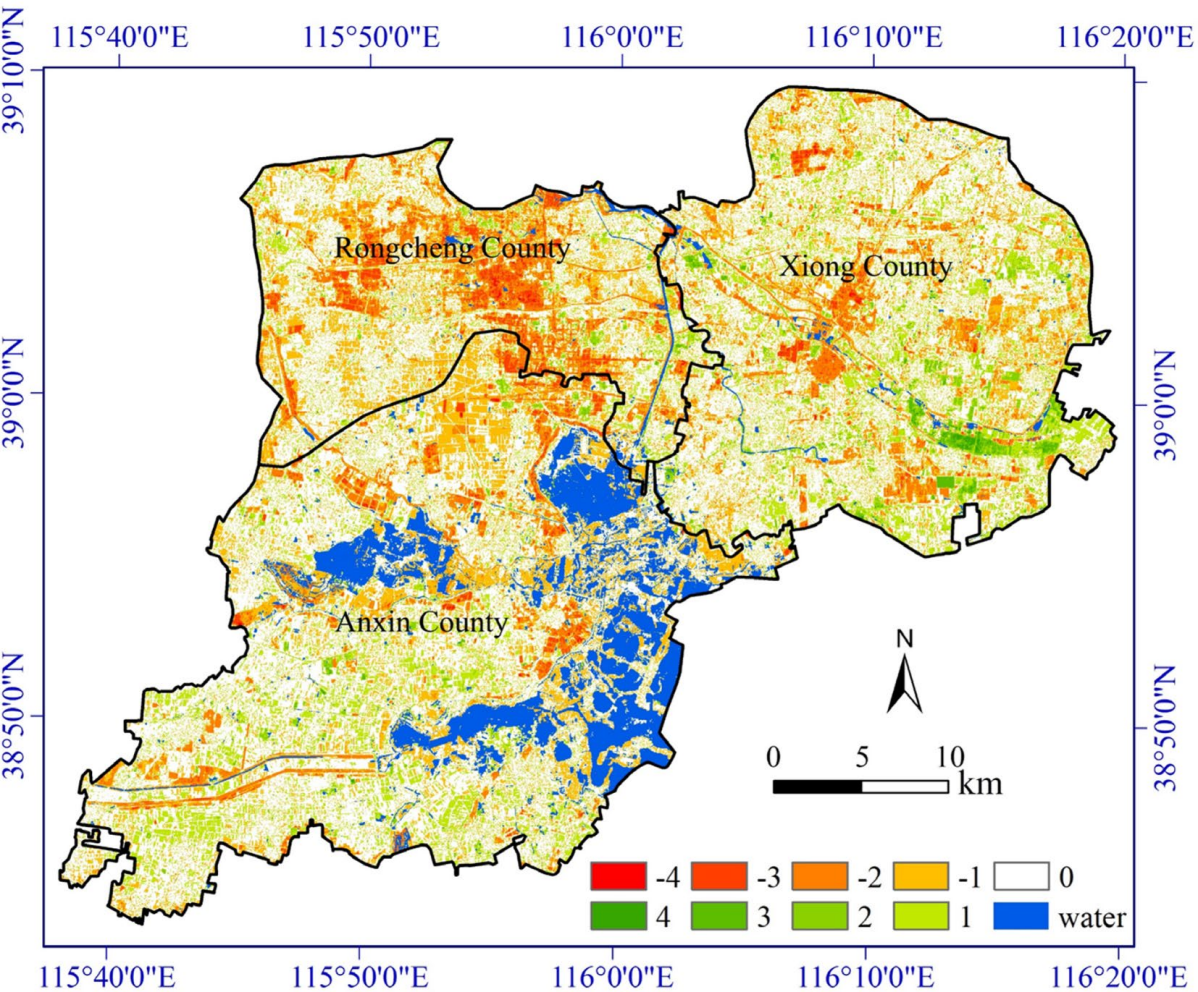
Change detection of RSEI level from 2014 to 2021. This map was produced using ArcGIS Version 10.7 (https://www.esri.com/en-us/arcgis/geospatial-platform/overview).

The main body of Xiong’an New Area consists of three counties: Xiong County, Rongcheng County, and Rongxin County. We conducted separate statistics for these three counties, and from Fig. 4, it can be observed that the RSEI in Rongcheng County has a significantly faster decline rate compared to the other two counties. Additionally, Fig. 5 reveals the change detection of RSEI level from 2014 to 2021, showing that over these seven years, there is a considerable proportion of areas in Rongcheng County where the RSEI level has declined by 2 levels or more. By referring to the relevant plans for Xiong’an New Area, it becomes evident that Rongcheng County was designated as the pioneering and starting area for the new area and thus was undergoing early-stage construction. This planning arrangement is a major policy factor contributing to a higher RSEI decline rate in Rongcheng County.

### Multi-scenario simulation of RSEI

From the above analysis of RSEI, it can be observed that Rongcheng County, as the pioneering and starting area for Xiong’an New Area, has significantly higher construction process and scale compared to Xiong County and Anxin County. The construction of Rongcheng County and the evolution of its EEQ may serve as the reference for the future development of the other two counties. Therefore, we conducted further multi-scenario simulation specifically focused on Rongcheng County.

**Fig. 6:**
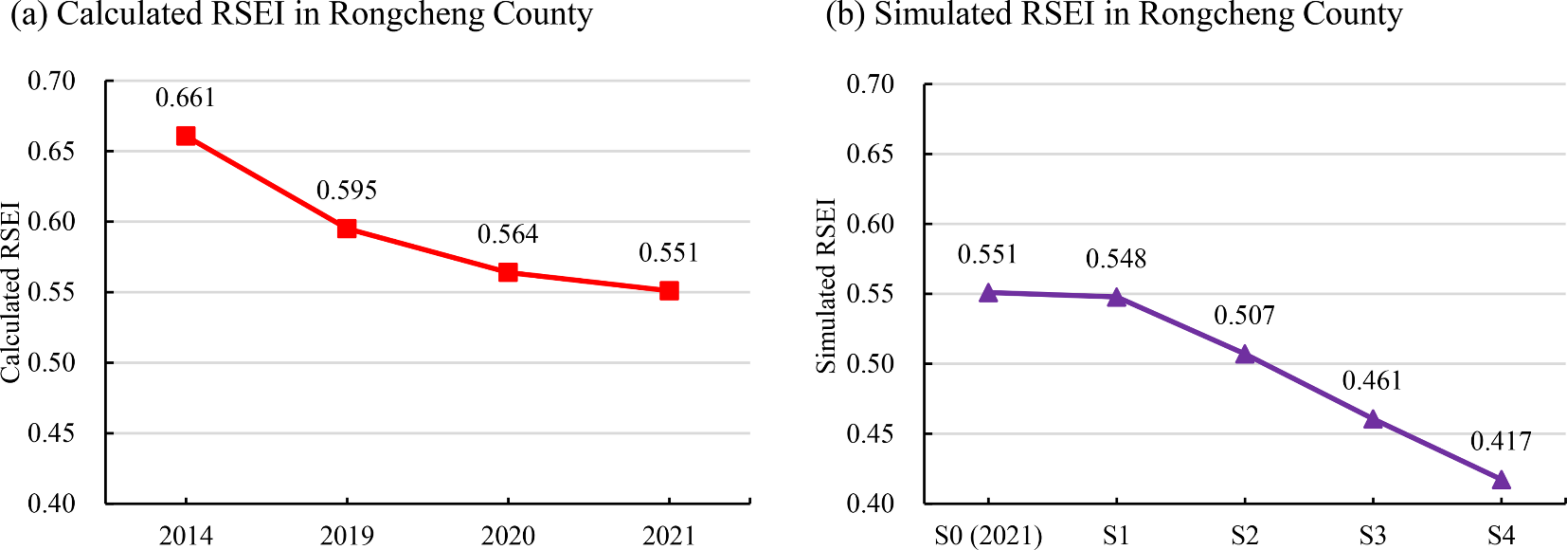
The actual trend of RSEI from 2014 to 2021 and the simulation results of RSEI for scenarios S1–S4 in Rongcheng County. (Note: Due to the presence of extensive bare soil resulting from crop harvesting in 2017, RSEI was significantly influenced by non-regional construction and therefore was not included in the overall trend shown in the figure (**a**).)

We constructed an XGBoost regression model using Rongcheng County’s 2021 RSEI as the dependent variable and five urban-rural construction features, i.e., NDVI_nor_, NDISI_nor_, PB, PopD, and BH, as independent variables. A total of 22,151 pixels were sampled for model construction. Through 5-fold cross validation, we determined the optimal combination hyperparameters of for seven important XGBoost model hyperparameters, i.e., eta = 0.129, gamma = 0.0309, max_depth = 8, min_child_weight = 6.76, subsample = 0.913, colsample_bytree = 0.83, and nrounds = 228. The model’s RMSE is 0.029. Furthermore, we utilized the constructed XGBoost regression model to perform the multi-scenario simulation on RSEI in Rongcheng County by progressively changing the urban-rural construction features by 5% (Fig. 2). The simulated RSEI values in scenarios S1–S4 are 0.548, 0.507, 0.461, and 0.417, respectively (Fig. 6b). Additionally, Fig. 6a presents that the actual trend of RSEI in Rongcheng County, which decreased from 0.661 in 2014 to 0.595 in 2019, and further to 0.564 in 2020 and 0.551 in 2021 (S0). It can be discovered that the results of the multi-scenario simulation exhibit a consistent linear trend with the actual change in RSEI.

**Fig. 7:**
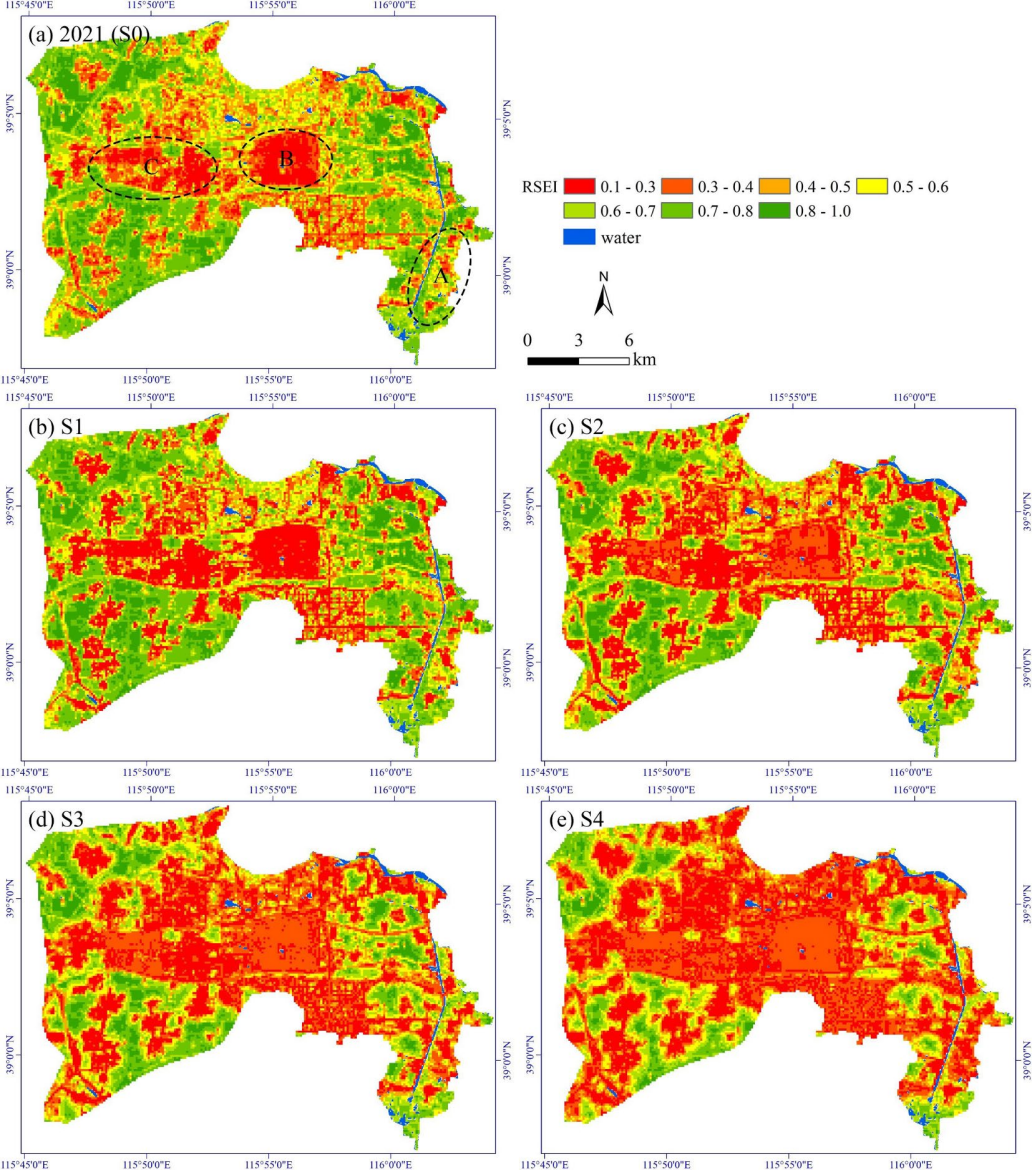
Spatial distribution of the multi-scenario simulation results for RSEI in Rongcheng County. This map was produced using ArcGIS Version 10.7 (https://www.esri.com/en-us/arcgis/geospatial-platform/overview).

Figure 7 shows the spatial distribution of the multi-scenario simulation results for RSEI in Rongcheng County. As expected, with the continuous expansion of urban and rural construction areas, the low-value areas of RSEI spread outward and replace the high-value ones. For example, in region A in 2021 (S0), it mainly consists of scattered rural residential areas (Fig. 1). With the evolution of simulated scenarios, the low-value areas of RSEI in this region expanded and merged, ultimately forming contiguous clusters of low RSEI values in S4. Additionally, we observed some noteworthy details in both reality and simulation results. For instance, in S0, the current situation of 2021, regions B and C in Rongcheng County are primarily developed areas (Fig. 1), with RSEI values in these regions mainly distributed between 0.1 and 0.3. However, as the simulation progressed, the RSEI in these two regions gradually increased, indicating that an improvement in EEQ. By S4, the RSEI in regions B and C, which were initially 0.1–0.3 in S0, have essentially shifted to a range of 0.3–0.4.

## Discussion

### The evolution of EEQ during the rapid urbanization in rural areas

This study utilized RSEI to quantitatively assessment the EEQ changes in Xiong’an New Area from 2014, before its establishment, to 2017, 2019 and 2020, during its initial establishment, and finally to 2021. The RSEI decreased from 0.648 in 2014 to 0.599 in 2021. During this study period, there was a substantial decrease in 2017 to 0.569. However, in 2019, the RSEI rebounded to 0.619, and then it steadily declined in 2020 and 2021, with values of 0.608 and 0.599 (Fig. 4). It can be observed that, despite a notable fluctuation in RSEI in 2017, the overall trend was one of gradual decline.

**Fig. 8:**
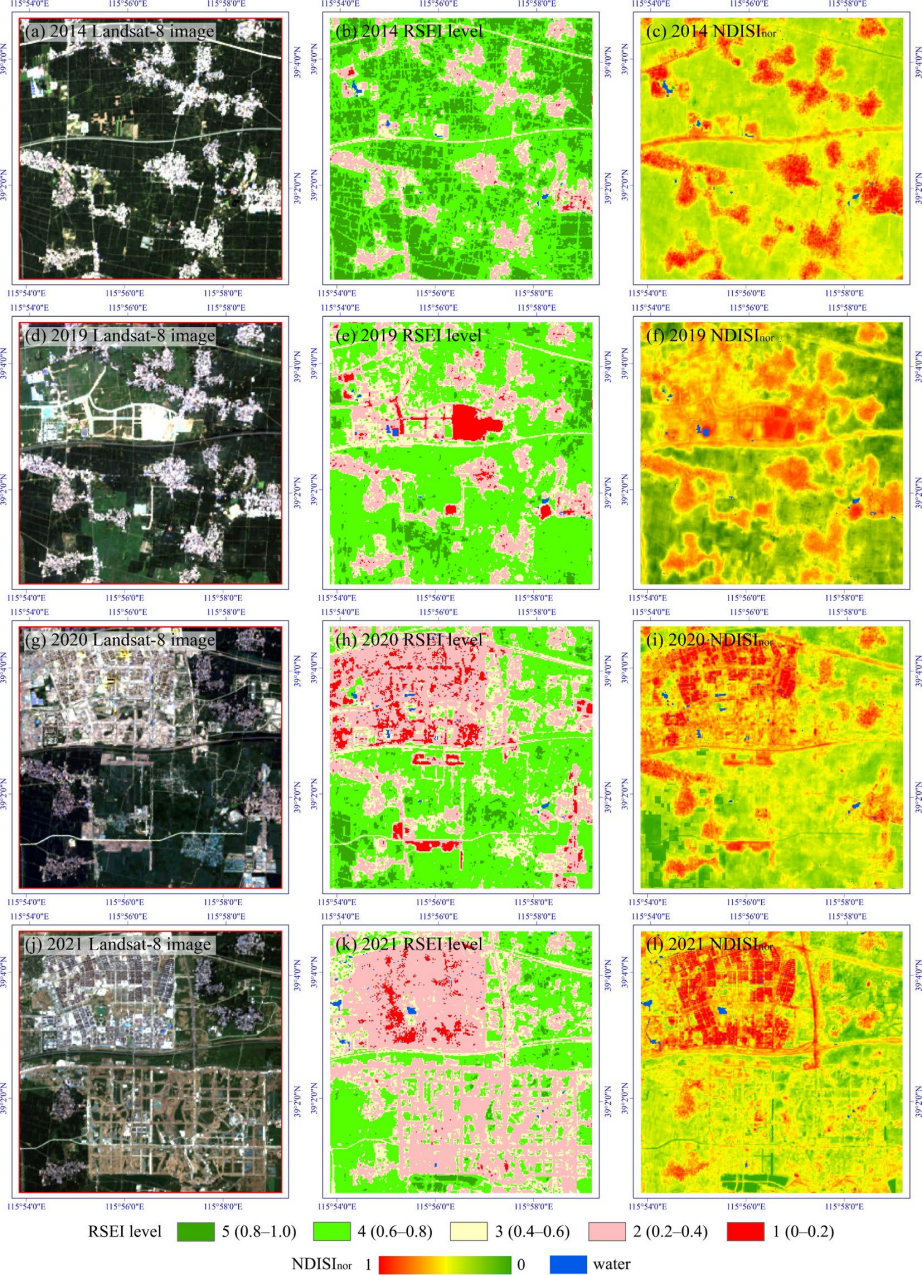
Ecological information related to the typical construction change area in Rongcheng County from 2014 to 2021. This map was produced using ArcGIS Version 10.7 (https://www.esri.com/en-us/arcgis/geospatial-platform/overview).

It can be seen from Fig. 8 that RSEI can effectively capture the comprehensive information of four indicators (greenness, wetness, heat, and dryness) to depict the entire process of the land surface transformation in rapidly developing areas. Figure 8b, e, h, k illustrate the construction process and corresponding RSEI level in a typical area of Rongcheng County from 2014 to 2021. It can be observed that during this construction process, RSEI does not strictly adhere to a high-to-low pattern of change, and the EEQ does not exhibit a linear progression from good to poor. Instead, the RSEI transitions from Level 4 or 5 for farmland to Level 2 for bare soil, and then decreases to Level 1 for impervious construction land. Moreover, as the functionality and the green infrastructure in the built-up areas continue to improve, its RSEI in those areas gradually increases to Level 2 again.

### The impact of urban-rural construction features on the multi-scenario simulation of RSEI

**Fig. 9:**
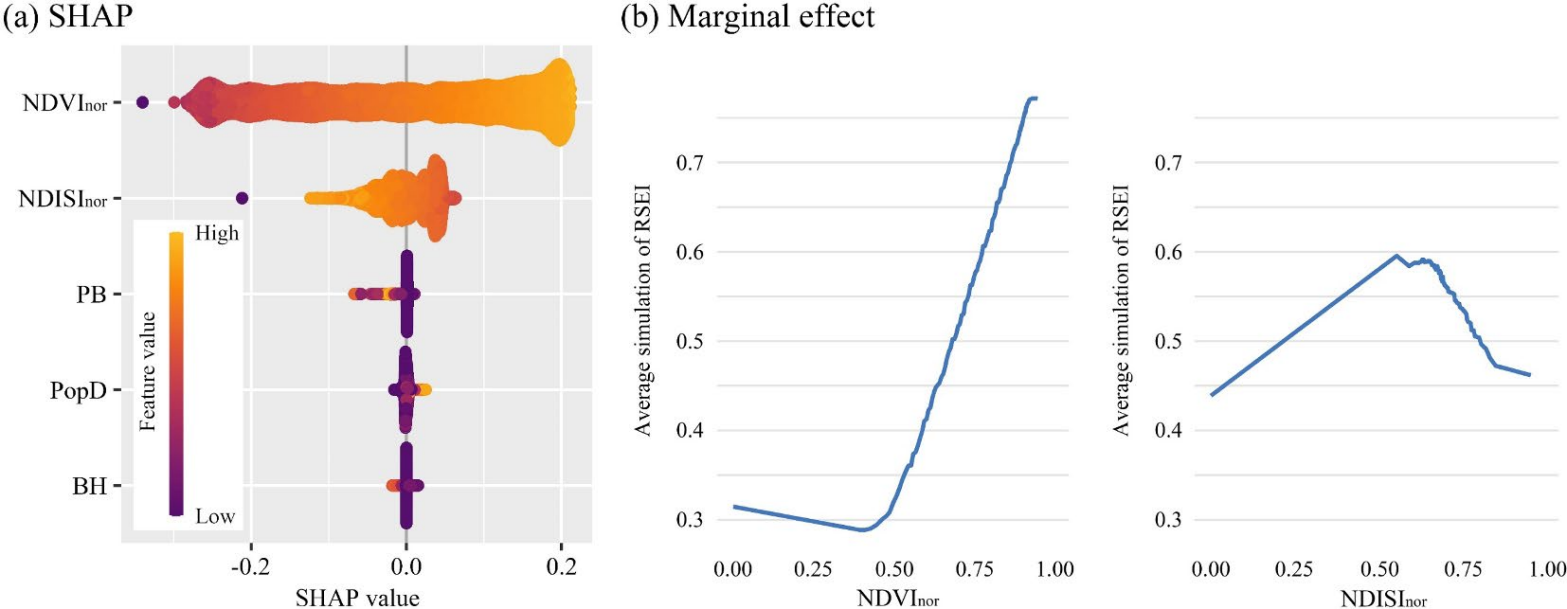
Analysis results of the urban-rural construction features in the XGBoost regression model.

In the multi-scenario simulation of RSEI in Rongcheng County, we discovered that as the urban-rural construction features continue to evolve, the EEQ of the currently existing construction areas will be improved in subsequent RSEI simulations (Fig. 7). To analyze this phenomenon, we evaluated the relative importance of these five construction features, i.e., NDVI_nor_, NDISI_nor_, PB, PopD, and BH, on simulated RSEI, using the XGBoost regression model. Figure 9a shows the SHAP analysis of the construction features employed in the XGBoost model, from which it can be seen that NDVI_nor_ has the most significant importance for simulated RSEI changes, followed by NDISI_nor_. The SHAP analysis also illustrates the directionality of the impact of construction features on RSEI. We can roughly discern that high NDVI_nor_ values have the most significant impact on improving RSEI, while low values have a counteracting effect on RSEI. However, it is difficult to accurately determine the directional effect of NDVI_nor_ and NDISI_nor_ on RSEI solely from Fig. 9a, which may suggest that their impact on simulated RSEI changes in non-monotonic.

From the marginal effects curve of NDVI_nor_ and NDISI_nor_ presented in Fig. 9b, it can be seen that they exhibit an approximate “U-shaped” curve and an inverted “U-shaped” curve, respectively, with significant turning points. Specifically, the NDVI_nor_ curve exhibits a turning point around 0.4. When NDVI_nor_ exceeds 0.4, there is a significant positive correlation between NDVI_nor_ and RSEI, indicating that richer vegetation correlates with better ecological quality. Conversely, when NDVI_nor_ is less than 0.4, the relationship between them is negative, and a smaller NDVI_nor_ can actually slightly improve the RSEI. Additionally, the marginal effects curve of NDISI_nor_ also exhibits a turning point, roughly located at 0.6. When NDISI_nor_ exceeds 0.6, an increase in impervious surface reduces regional EEQ. In Contrast, when NDISI_nor_ is less than 0.6, it is unexpected that an increase in impervious surface would improve EEQ. Observing Fig. 8b, e, h, k, it becomes evident that RSEI values in the impervious land for construction preparation are the lowest, classified as RSEI Level 1, followed by bare soil and building construction area, which are classified as RSEI Level 2. Moreover, from Fig. 8c, f, i, l, although both bare soil and building construction areas belong to Level 2, the building construction areas exhibit a significantly higher NDISI_nor_ than bare soil. Thus, we speculated that the existence of the turning point in the marginal effects curve is mainly due to the large-scale construction activities in rural areas, where land cover has undergone a transformation from farmland or natural elements cover to bare soil, followed by the transformation into impervious lands for construction preparation, and finally to building construction areas and built-up areas. Thoughout this developmental process, the regional EEQ, as characterized by RSEI, does not monotonically decrease. Rather, it significantly deteriorates in the initial stages of construction, and then shows a certain degree of recovery in the later stages of development.

In our study, we discovered an inverted “U-shaped” curve in the relationship between NDISI_nor_ and simulated RSEI (Fig. 9b). Numerous studies have demonstrated the existence of this non-linear relationship between urbanization and EEQ^48,49^. For instance, Liu et al.^5^ found a “U-shaped” curve between human activity intensity and EEQ in their study of the spatiotemporal relationship between humans and nature on the Qinghai-Tibet Plateau. Their research revealed a turning point in the process of urbanization, Making a transition from negative to positive of human activity on EEQ. Some scholars argued that in the regions with limited urban development , urbanization can concentrate the population in urban built-up areas, thereby reducing the dispersion of human activities and decreasing the interference on EEQ caused by activities such as farming and grazing^50^. Moreover, some researchers have employed the Environmental Kuznets Curve (EKC) to describe the relationship between economic development and EEQ, Which also reveals a “U-shaped” curve between regional development and EEQ^51–53^. Additionally, Xu et al.^54^ added the indicator for abundance index for land cover type to the four RSEI indicators (greenness, wetness, heat, and dryness) and constructed RSEI-2. Their study also observed the “U-shaped” curve between urbanization and the coupling coordination degree of EEQ. It is worth noting that, although our study was able to demonstrate the existence of a “U-shaped” relationship and its turning point, we cannot definitively consider the values of NDVI_nor_=0.4 and NDISI_nor_=0.6 obtained in the current marginal effect curve as the precise key turning points in their relationship with EEQ. It is because the construction in Xiong’an Area is still ongoing. However, even so, we may speculate that the relationship between humans and nature, or urbanization and EEQ may be not absolute zero-sum game, and win-win situations can be achieved under certain conditions^52^.

From Fig. 9a, it can be seen that besides the two urban-rural construction features, NDVI_nor_ and NDISI_nor_, which have a significant relative importance in determining RSEI, PB, PopD, and BH have relatively low influence on RSEI changes. This may be attributed to the unique characteristics of rural areas in China. In rural China, building clusters exhibit a combination of small-scale concentration and large-scale dispersion. Within individual rural settlements, the PB is relatively high, but overall, the PB is low. Additionally, buildings in rural areas are generally low-rise, and there is a minimal fluctuation of BH within a localized area. As for PopD feature, the majority of young people in rural China migrate to more developed cities for employment, resulting in generally low PopD in rural areas. However, in recent years, with the continuous construction and development of Xiong’an New Area, it has attracted some locals as well as people from other regions to work in Xiong’an, though the number of individuals involved remains limited during the initial stages of development in the new area. Xu et al.^12^ also observed a weak inverse relationship between PopD and RSEI. The analysis yielded an *R*^2^ value of 0.0427 (*p* < 0.01), indicating a very low degree of explanatory power. However, an interesting result was discovered when they introduced a new variable called impervious surface area-related population density (IPD), which quantifies the population per unit area of impervious surface. They found a remarkably strong correlation between IPD and RSEI, with an *R*^2^ value of 0.9996 (*p* < 0.01).

### Implications for urban and rural planning and management

In the process of urban-rural development in the rural areas, the substitution of the natural elements such as forests, wetlands, and water bodies, as well as artificial elements such as cultivated lands, farmland, and villages, is inevitable and leads to a decline in EEQ. However, if scientific planning and guidance can be implemented based on the concept of ecological priority, the impact of construction on EEQ can be minimized. According to the industrial development positioning for Xiong’an New Area, the first step is to consolidate the scattered rural settlements within the new area, making the residential areas of local villagers more concentrated and improving the supporting facilities for their lives. Moreover, the excess villages will be vacated and restored to farmlands, forests, and wetlands. Currently, Rongcheng County serves as the pioneering and starting area, taking the lead in large-scale construction, while later, Anxin County, with a large agricultural industry, will be designated as a key development area for green and ecological agriculture. It aims not only to preserve a large area of arable lands but also integrate high-tech industries and build modern agriculture facilities, achieving integration of ecology and industry.

## Conclusions

Xiong’an New Area is state-level new area officially established in 2017. This study quantitatively assessed the EEQ changes in Xiong’an New Area from 2014 to 2021 using RSEI. Moreover, we utilized the XGBoost machine learning algorithm to simulate the RSEI across multiple construction scenarios in Rongcheng County, the pioneering and starting area of the new area. The main conclusions are summarized as follows: (1) With the large-scale construction in Xiong’an New Area, the regional EEQ shows a downward trend from 0.648 in 2014 to 0.599 in 2021. The primary driver behind the decline in RSEI was the conversion of agricultural land and natural surface into construction areas. (2) Rongcheng County experienced a faster decline in RSEI compared to Xiong County and Anxin County. By using XGBoost regression model and setting scenarios with a 5% progressive change in the urban-rural construction features, the simulated RSEI results of Rongcheng County, in the progressive scenarios S1–S4, were 0.548, 0.507, 0.461, and 0.417, respectively. These simulated results exhibited a consistent linear trend with the actual change in EEQ. (3) The large-scale construction in rural areas in China involves land surface cover changes from agricultural land and natural surface to bare soil, then from bare soil to impervious land for construction preparation, and finally to building construction areas and built-up areas. During this process, the EEQ in local areas exhibits an initial significant decline followed by slight recovery. (4) The marginal effects of urban-rural construction features for simulated RSEI revealed an inverted “U-shaped” curve in the relationship between urbanization and EEQ. This indicates that the relationship between humans and nature or urbanization and EEQ may not be absolute a zero-sum game, as it can achieve a win-win situation under certain suitable conditions. Our findings can provide significant insights for regional construction and offer a scientific basis for the sustainable development of ecological environment under rapid urbanization.

## Data Availability

The Landsat-8 image data can be obtained from the USGS EarthExplorer online platform (https://earthexplorer.usgs.gov/). The population density data can be obtained from WorldPop website (https://www.worldpop.org/). The China building rooftop area dataset can be accessed at https://zenodo.org/record/7500612. The Chinese building height dataset can be accessed at https://zenodo.org/record/7064268#.ZEQM-vxByUm.
